# A84 THE USE OF PHYTASE TO ENHANCE DIETARY IRON AND ZINC ABSORPTION - A SCOPING REVIEW

**DOI:** 10.1093/jcag/gwae059.084

**Published:** 2025-02-10

**Authors:** S Hirota, N Adamidi, T Chondrou, D Lygouras, O Androutsos, V Svolos

**Affiliations:** Physiology & Pharmacology, University of Calgary, University of Calgary, Calgary, AB, CA, academic, Calgary, AB, Canada; Lab of Clinical Nutrition And Dietetics, Department of Nutrition and Dietetics, School of Physical Education, Sport Science And Dietetics, University of Thessaly, Volos, Greece; Lab of Clinical Nutrition And Dietetics, Department of Nutrition and Dietetics, School of Physical Education, Sport Science And Dietetics, University of Thessaly, Volos, Greece; Department of Computer Science, Democritus University of Thrace, Xanthi, Greece; Lab of Clinical Nutrition And Dietetics, Department of Nutrition and Dietetics, School of Physical Education, Sport Science And Dietetics, University of Thessaly, Volos, Greece; Lab of Clinical Nutrition And Dietetics, Department of Nutrition and Dietetics, School of Physical Education, Sport Science And Dietetics, University of Thessaly, Volos, Greece

## Abstract

**Background:**

Phytic acid is abundant in plant-based diets and acts as a micronutrient inhibitor for humans and non-ruminant animals. Phytases are enzymes that break down phytic acid, releasing micronutrients and enhancing their bioavailability, particularly iron and zinc. Deficiencies in iron and zinc are common in patients with the inflammatory bowel diseases and in those in developing countries consuming phytic acid-rich diets.

**Aims:**

This literature review aimed to summarize findings from human intervention studies on the interactions between phytic acid, phytase and micronutrient bioavailability.

**Methods:**

An extensive PubMed search (01/01/1990 to 08/02/2024) was conducted using MeSH terms (phytic acid, phytase, IP6, “inositol hexaphosphate,” micronutrient, magnesium, calcium, iron, zinc). Eligible studies included human intervention trials investigating the bioavailability of micronutrients following a) phytase supplementation, b) consumption of phytic acid-rich foods, or c) consumption of dephytinised foods. In-vitro, animal, cross-sectional, and non-English studies were excluded.

**Results:**

3055 articles were identified. After title and full-text review 40 articles were eligible. Another 2 were identified after cross-checking reference lists from included papers, resulting in 42 included articles. Most studies exploring the efficacy of exogenous phytase (9 of 11, 82%) or the efficacy of food dephytinisation (11 of 14, 79%) presented augmented iron and zinc bioavailability. In agreement to this, most phytic acid-rich food feeding studies (13 of 17, 77%) showed compromised iron and zinc bioavailability.

**Conclusions:**

Strong evidence supports decreased iron and zinc bioavailability in phytic acid-rich diets and the improvement potential with phytase interventions. Further studies are needed in larger populations.

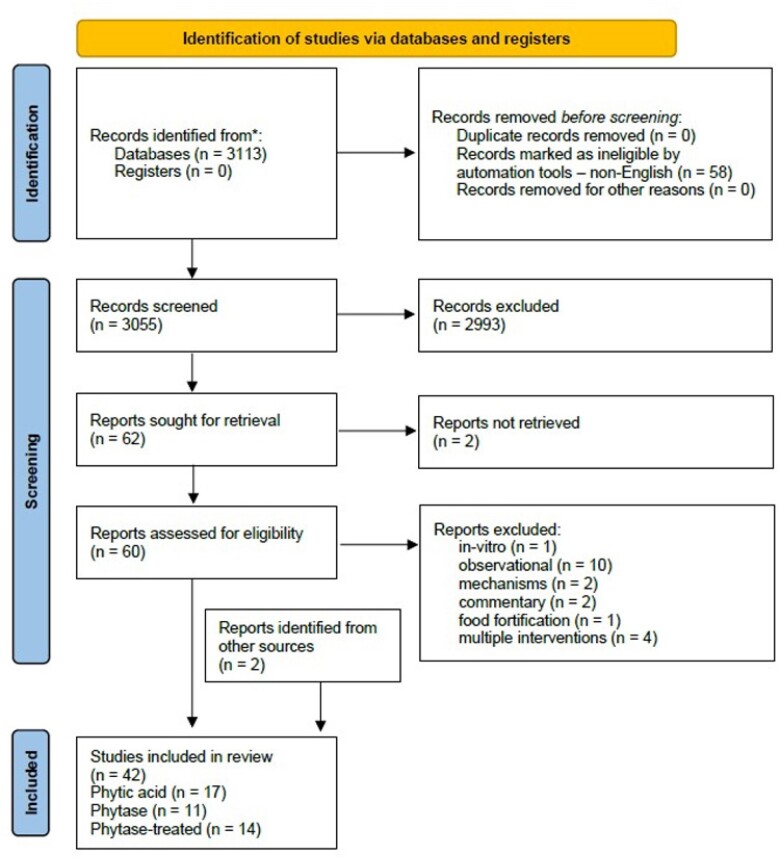

**Funding Agencies:**

None

